# Comparison of Whiskbroom and Pushbroom darkfield elastic light scattering spectroscopic imaging for head and neck cancer identification in a mouse model

**DOI:** 10.1007/s00216-021-03726-5

**Published:** 2021-11-19

**Authors:** Miriam C. Bassler, Mona Stefanakis, Inês Sequeira, Edwin Ostertag, Alexandra Wagner, Jörg W. Bartsch, Marion Roeßler, Robert Mandic, Eike F. Reddmann, Anita Lorenz, Karsten Rebner, Marc Brecht

**Affiliations:** 1 Process Analysis and Technology (PA&T), Reutlingen University, Alteburgstr. 150, 72762 Reutlingen, Germany; 2 Institute of Physical and Theoretical Chemistry, University of Tübingen, Auf der Morgenstelle 18, 72076 Tübingen, Germany; 3 Institute of Dentistry, Barts and the London School of Medicine and Dentistry, Queen Mary University of London, London, UK; 4 Department of Neurosurgery, Philipps University Marburg, Baldingerstraße, 35033 Marburg, Germany; 5 Department of Pathology, Philipps University Marburg, Baldingerstraße, 35033 Marburg, Germany; 6 Department of Otorhinolaryngology, Philipps University Marburg, Baldingerstraße, 35033 Marburg, Germany

**Keywords:** Mie elastic light scattering spectroscopy, Chemometrics/statistics, Clinical/biomedical analysis, Head and neck cancer, Mouse tumor model, Microspectroscopy

## Abstract

The early detection of head and neck cancer is a prolonged challenging task. It requires a precise and accurate identification of tissue alterations as well as a distinct discrimination of cancerous from healthy tissue areas. A novel approach for this purpose uses microspectroscopic techniques with special focus on hyperspectral imaging (HSI) methods. Our proof-of-principle study presents the implementation and application of darkfield elastic light scattering spectroscopy (DF ELSS) as a non-destructive, high-resolution, and fast imaging modality to distinguish lingual healthy from altered tissue regions in a mouse model. The main aspect of our study deals with the comparison of two varying HSI detection principles, which are a point-by-point and line scanning imaging, and whether one might be more appropriate in differentiating several tissue types. Statistical models are formed by deploying a principal component analysis (PCA) with the Bayesian discriminant analysis (DA) on the elastic light scattering (ELS) spectra. Overall accuracy, sensitivity, and precision values of 98% are achieved for both models whereas the overall specificity results in 99%. An additional classification of model-unknown ELS spectra is performed. The predictions are verified with histopathological evaluations of identical HE-stained tissue areas to prove the model’s capability of tissue distinction. In the context of our proof-of-principle study, we assess the Pushbroom PCA-DA model to be more suitable for tissue type differentiations and thus tissue classification. In addition to the HE-examination in head and neck cancer diagnosis, the usage of HSI-based statistical models might be conceivable in a daily clinical routine.

**Graphical abstract:**

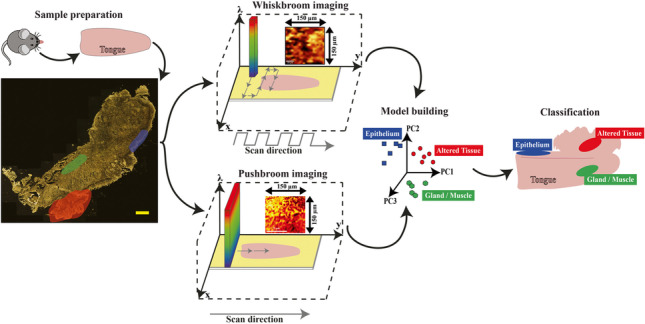

**Supplementary Information:**

The online version contains supplementary material available at 10.1007/s00216-021-03726-5.

## Introduction

Microspectroscopic imaging is a powerful tool to investigate biological materials of any kind and helps to reveal their structure, functionality, and purpose within the organism. It correlates the spectroscopic data with the microscopic image and thus allows for a spatial assignment of spectral information. Important biological applications for microspectroscopic imaging, such as Raman, fluorescence, or infrared (IR) imaging, are the analysis of cell systems and tissues [1, 2], particularly in cancer research and diagnosis [3].

Despite intense research and therapy development, cancer still belongs to one of the most threatening diseases humankind is suffering. In 2018, approximately 18.1 million new cancer cases were estimated worldwide with a higher incidence in lung cancer, female breast cancer, and colorectal cancer [4], closely followed by head and neck squamous cell carcinomas (HNSCC), as the sixth most common cancer type [5]. HNSCC encompass a variety of tumors originating in the lip, oral cavity, hypopharynx, oropharynx, nasopharynx, or larynx. Oral squamous cell carcinomas (OSCC), a subset of HNSCC, account for 355,000 new cases annually worldwide [6] with a 5-year survival rate of 50% [7]. In recent studies, significant inter-tumoral heterogeneity was observed by histopathology, reflecting the tumor site of origin, proliferation, the grade of differentiation, depth of invasion, and degree of inflammation [8]. Oral cancer screening is inevitable for early detection and early treatment of tumors, which could considerably improve survival rates.

Early detection of HNSCC requires sensitive identification and localization methods, able to measure small cell and tissue changes. So far, hematoxylin–eosin staining (HE-staining) represents the gold standard in histopathology to recognize head and neck (HN) lesions in several stages during carcinogenesis. Additional early detection tools for HNSCC comprise spectroscopic technologies like narrowband imaging (NBI) [9], Raman [10], and fluorescence spectroscopy [11]. One spectroscopic technique demonstrated exceptional suitability to early detect preneoplastic variations during colorectal cancer genesis, which is elastic light scattering spectroscopy (ELSS) [12]. Already 2 weeks prior to the first evidence of malignant tissue alteration, marked changes were detectable by measuring nano- and microscale architectures of the colonic tissue with elastic light scattering (ELS) [12]. On the cellular level, ELSS elucidates morphological features such as size distribution of cells and nuclei or the degree of nuclei pleomorphism and hyperchromasia [13]. For the morphological tissue characterization and differentiation, great achievements with ELSS could not only be generated in colorectal cancer [14], but also in breast cancer [15], skin cancer [16], brain tumors [17], and most importantly in HNSCC [18]. Recently, our group demonstrated to distinguish formalin-fixed brain tumor tissues with a varying degree of malignancy by optical spectroscopy, including ELSS [19].

The implementation of ELSS as an imaging method allows for the combination of spatial (x, y-direction) with spectral information (*λ*-direction). This results in a 3D data matrix called a hypercube [20, 21]. The hypercube can be evaluated in two ways: image planes can either be extracted at certain wavelength bands or the whole spectrum of one x, y-coordinate or pixel is used within the image plane [22]. Various studies of cancer diagnosis with HSI were accomplished [23–25]. HSI has yet been investigated in terms of its overall suitability in cancer detection and diagnosis using different cancer and sample types, spectral ranges, light sources, acquisition modes, or evaluation algorithms [21]. Most groups so far concentrated on the successful classification of different tissue or cell types to make it useable in cancer surgery. Our study, however, aims to investigate, for the first time, ELSS with two varying HSI detection principles, which are Whiskbroom and Pushbroom imaging, in order to compare their ability to detect ELS of predefined tissue samples. We want to verify whether the time-consuming point-by-point measurement of the Whiskbroom imaging is more suitable for ELS detection than the line-scanning system of a Pushbroom imager since both imaging methods exhibit different lateral and spectral resolution capabilities [21]. With respect to data acquisition, we modified our instrumental setups by installing a darkfield (DF) illumination pathway according to Ostertag et al. [26, 27]. Our adaption consequently enables darkfield elastic light scattering (DF ELS) as a non-contact microspectroscopic imaging modality. For a better understanding and comparability of both scanning techniques, advantages and disadvantages are summarized in Table 1.

**Table 1:** Advantages and disadvantages of Whiskbroom (column 2) and Pushbroom (column 3) imaging listed by several criteria (column 1). The following overview should emphasize the strength of each individual imaging method and point out customized features of the setups used in this study. Column 4 lists the references to each feature

Criteria	Whiskbroom imaging	Pushbroom imaging	References
Spectral resolution	High	High	[20, 21], customized
Spatial resolution	High	High	[28]
Scanning speed	Time-consuming	Fast	[21]
Field of measurement	Restricted (150 µm × 150 µm) (by piezo table)	Large (340 µm × several mm)(x-direction × y-direction)	Customized
Spectrometer entrance	Pinhole-based	Slit-based	Customized
Spatial aliasing	No	Yes	[21], customized
Acquisition mode	Off-line	On-line/in-line	[20]
Splitting of light	uniformly high efficiency due to dispersive elements	Uniformly high efficiency due to dispersive elements	[28]
Costs	High ≥ 100,000 €	Low ≤ 100,000 €	[28], customized
Hardware	Complex	Complex	[28]

As a proof-of-concept study, DF ELS Whiskbroom and Pushbroom imaging are applied on a HNSCC mouse model analyzing longitudinal-cut tissue sections of mouse tongues. The obtained spectral images are analyzed using multivariate data analysis (MVA). By combining a principal component analysis (PCA) and Bayesian discriminant analysis (DA), we generate two statistical models of the tongue tissue data based on both HSI detection techniques and compare if one of the models achieves better results in discriminating different tongue tissue types. Based on this discrimination, we derive whether noticeable differences between Whiskbroom and Pushbroom imaging appear. Finally, the performance of both statistical models is determined and verified regarding their ability to correctly classify model-unknown ELS spectra of varying tissue types. An additional comparison of the statistical model performance with classical HE histopathology is accomplished. If proven reliable, the statistical models could help to provide diagnostic information for physicians during surgery.

## Materials and methods

### Mouse model for OSCC and carcinogenesis

For this proof-of-concept study, we used an autologous mouse model of OSCC. A total amount of four different mice was investigated. Mice were subdivided into two control mice and two mice developing tumors. Control mice (mice A and B) exhibit an intact tissue structure. Therefore, mice A and B mainly provide healthy epithelium specimens as well as glandular and muscle tissue. Mice with dysplastic alterations (mice C and D) display several stages of tissue modification like hyperplastic and dysplastic areas or invasive squamous cell carcinomas (SCC). Modified tissue, mainly composed of invasive SCC, was derived from mouse C. Altered tissue specimens of mouse D function as prediction areas for the statistical model validation. Mice were maintained on the C57Bl/6 N genetic background.

Tumors are induced by chronic oral administration of the water-soluble carcinogen 4-nitroquinoline-1-oxide (4NQO) [8, 29, 30] that mimics the alterations caused by tobacco mutagens. 4NQO forms DNA adducts, causing substitution of adenosine for guanosine, and induces intracellular oxidative stress resulting in mutations and DNA strand breaks [31]. Additionally, 4NQO is known to induce point mutations in *HRas* with subsequent loss of heterozygosity [32], upregulation of *EGFR* [33], p53 mutations [8], and reduced expression of the cell cycle inhibitor p16 [33]. These effects are similar to the genetic alterations induced by tobacco carcinogens [8, 33]. As in human OSCC, invasive tumors are preceded by epithelial hyperplasia and dysplasia [8].

**Fig. 1:**
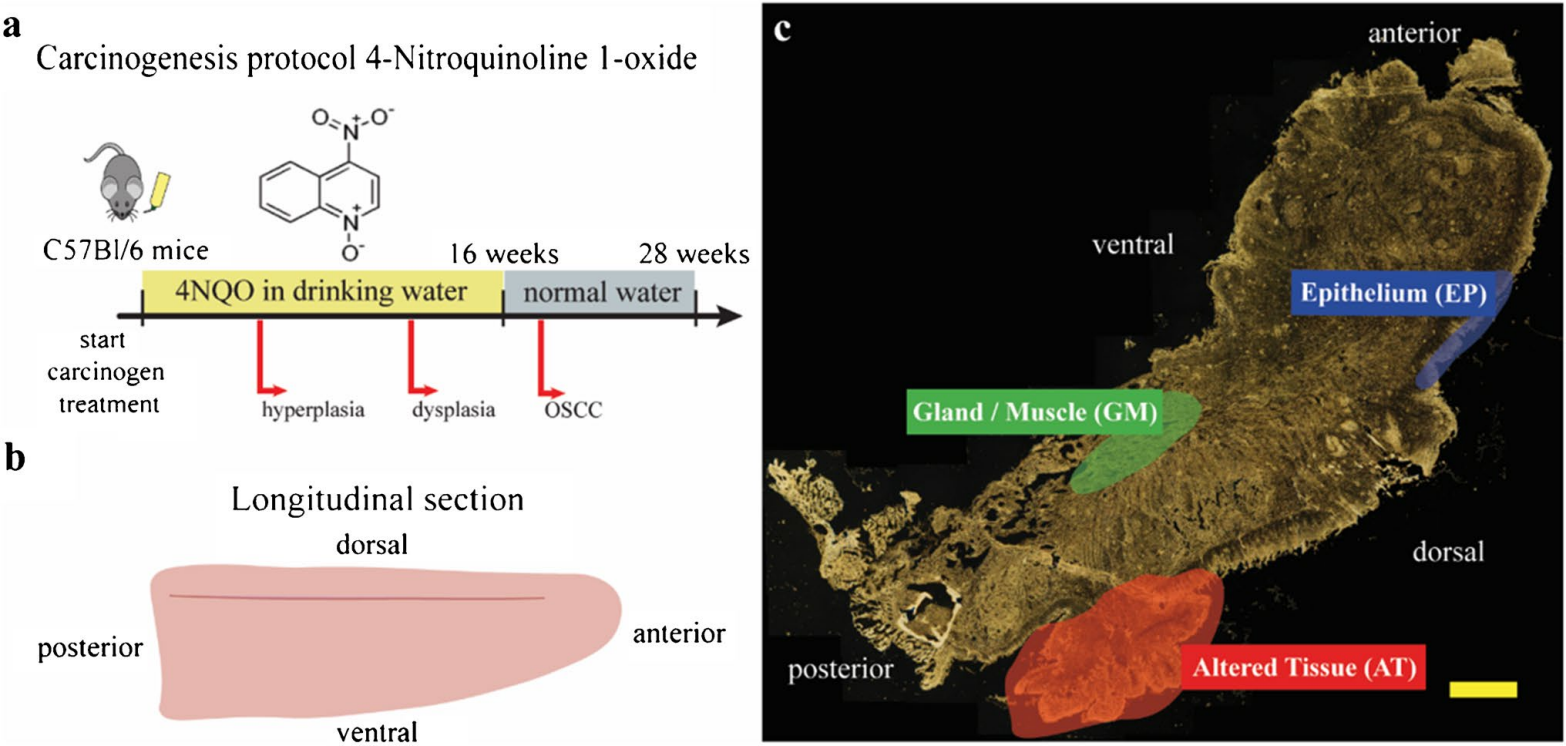
Principle of tumor induction in C57Bl/6 N mice. (a). At the beginning of carcinogen treatment, mice were administered the tumorigenic compound 4NQO via the drinking water over a total interval of 16 weeks (a). Throughout this treatment, mice developed various carcinogenic stages including hyperplasia after 6 weeks, dysplasia after 12 weeks, and oral squamous cell carcinoma (OSCC) after 18 weeks. The administration of 4NQO was finished in week 16 and mice were supplied with normal drinking water at that point. Although the 4NQO administration stopped, OSCC lesions emerged. The anatomical position and direction of longitudinal tongue cross sections is elucidated in b. An exemplary darkfield (DF) image of a mouse tongue is shown in c. Different tissue regions, such as gland/muscle (green), epithelium (blue), and altered tissue (red), were assigned after a histopathological evaluation of a corresponding HE-stained tissue section (yellow scale bar: 1000 µm)

Mice from both genders were maintained on the C57BL/6 N genetic background and were housed under a 12-h light/12-h dark cycle, at temperatures of 20–24 °C with 45–65% humidity. Starting weights of the mice ranged between 25 g and 32 g. 4NQO (Sigma, diluted to 100 µg/mL in water) was administered in the drinking water and changed once a week for 16 weeks (Fig. 1a). After that period, C57Bl/6 N mice were given drinking water without 4NQO. Mice were maintained with regular mouse chow and water (± 4NQO) *ad libitum*. Once a week, 4NQO-treated mice were sedated with inhaled isoflurane and the oral cavities were screened for lesions (hyperplasias, dysplasias, and SCCs) [8, 30]. All animal procedures were subject to institutional ethical review and approved by the UK Home Office (in accordance with UK law, Animals Scientific Procedures Act 1986) at King’s College London prior to commencement (Project license number 70/8474). We adhere to ARRIVE guidelines as set out by the NC3Rs.

Tongue tissues were harvested and embedded in OCT (optimal cutting temperature compound, VWR). Sequential cross sections were cut using a cryostat (CryoStar NX50, ThermoFisher) at 10 µm thickness and post-fixed in 3.7% paraformaldehyde/PBS pH 7.4 for 15 min, washed twice in PBS and air-dried before staining. All murine tongues were cut equally in a longitudinal cutting direction. For DF ELS imaging, tissue sections were transferred to gold-coated (BioGold™ 100 nm coat thickness, Thermo Scientific™) microarray slides. Comparable tissue cross sections were additionally placed on glass objective slides and HE-stained by conventional methods. By HE-staining, lesions were identified and classified macroscopically and microscopically. We found lesions on the dorsal and ventral tongue (Fig. 1b, c) and some animals presented more than one lesion. HE-images were acquired using a Hamamatsu slide scanner and analyzed using NanoZoomer software (Hamamatsu).

Tumor grading was assessed according to the presence of the following criteria: tumor cell crowding, degree of keratinization, exophytic or invasive growth, scattered mitotic figures, and nuclear atypia [34]. All histological assessments were performed by a pathologist blinded to the study groups and 4NQO treatment conditions.

Owing to the great heterogeneity of tumors and modified tissues [35], we refrain from distinguishing several stages of carcinogenesis, but define all types of tumor alterations, including hyperplasia, dysplasia, and invasive SCC, as altered tissue (AT) (Fig. 1c). The healthy counterpart consists of the epithelium (EP) and a mixture of glandular and muscle tissue (GM) (Fig. 1c), summarized as stroma.

### Workflow of the HSI PCA-DA model development and validation

**Fig. 2:**
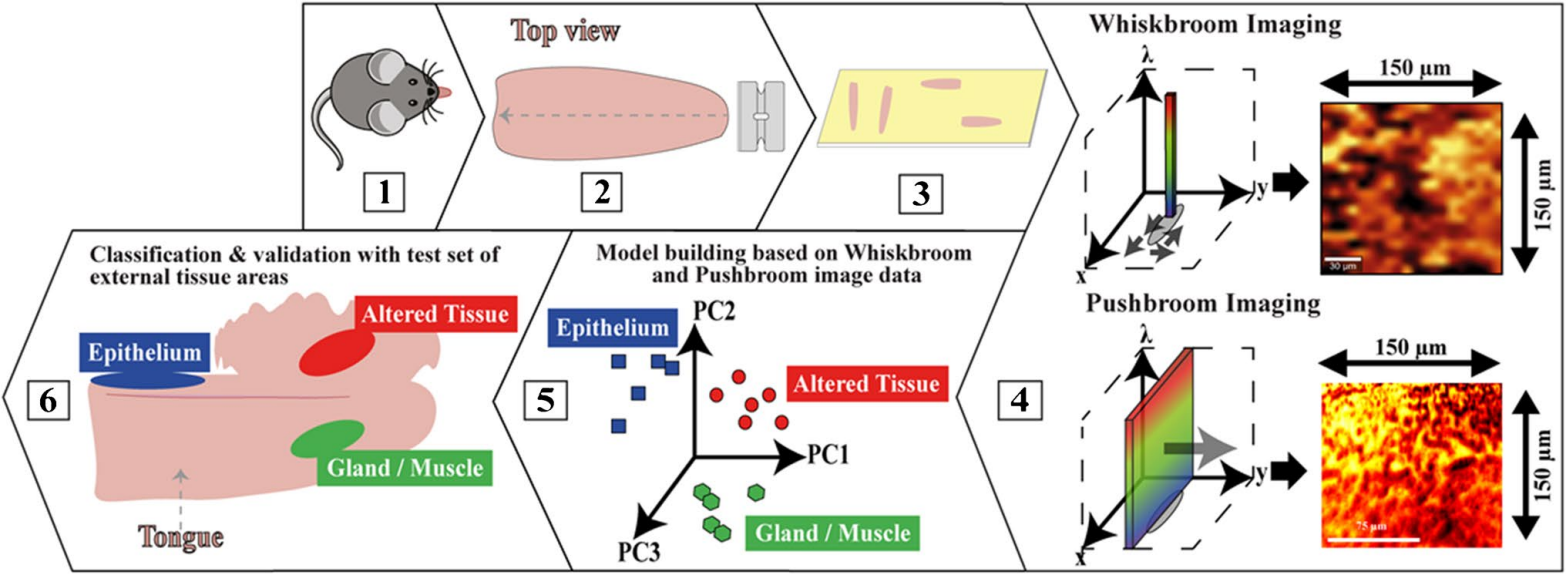
Schematic illustration of the HSI PCA-DA model development and validation workflow applied for an OSCC mouse model. In 1: C57Bl/6 N mice breeding. In 2: mice tongues harvesting and sequential cross section cutting in longitudinal direction. In 3: tongue cross sections were transferred to gold-coated microscope slides. In 4: DF ELS Whiskbroom and Pushbroom imaging of predefined tissue regions for EP, GM, and AT. In 5: PCA-DA model development for both techniques. In 6: validation of the Whiskbroom and Pushbroom PCA-DA model with a testing set of different tissue spectra. The model predictions are illustrated as colored ellipses for each tissue type

To create lingual tissue classification models, consecutive working steps need to be realized, starting from mouse breeding, drug-induced carcinogenesis protocol, tissue harvesting, and tongue preparation to the final statistical evaluation of spectroscopic image data. This holistic approach is summarized in Fig. 2.

As displayed in Fig. 2, C57Bl/6 N mice were bred for a total of 28 weeks and treated with 4NQO (1). After treatment, mice tongues were harvested and a sequential cross section cutting of the tongues in a longitudinal direction was performed (2). The tongue and microtome blade (2) are schematically shown in top view. Different profile sections of each mouse were prepared on individual gold-coated microscope slides (3). ELS DF Whiskbroom and Pushbroom imaging was accomplished on histopathologically predefined tissue regions of EP, GM, and AT (4). For Whiskbroom imaging, measuring areas were kept at 150 µm × 150 µm whereas measuring regions for the Pushbroom imaging can differ among images. An exemplary Pushbroom imaging region of 150 µm × 150 µm is depicted in (4). PCA-DA models were developed for both techniques in order to enable a distinct differentiation of all three tissue types (5). The Whiskbroom and Pushbroom PCA-DA models were tested by predicting model-unknown datasets of GM, EP, and AT (6). The model predictions are illustrated as colored areas for each tissue type (6).

### ELS spectra acquisition in Whiskbroom and Pushbroom imaging modes

**Fig. 3:**
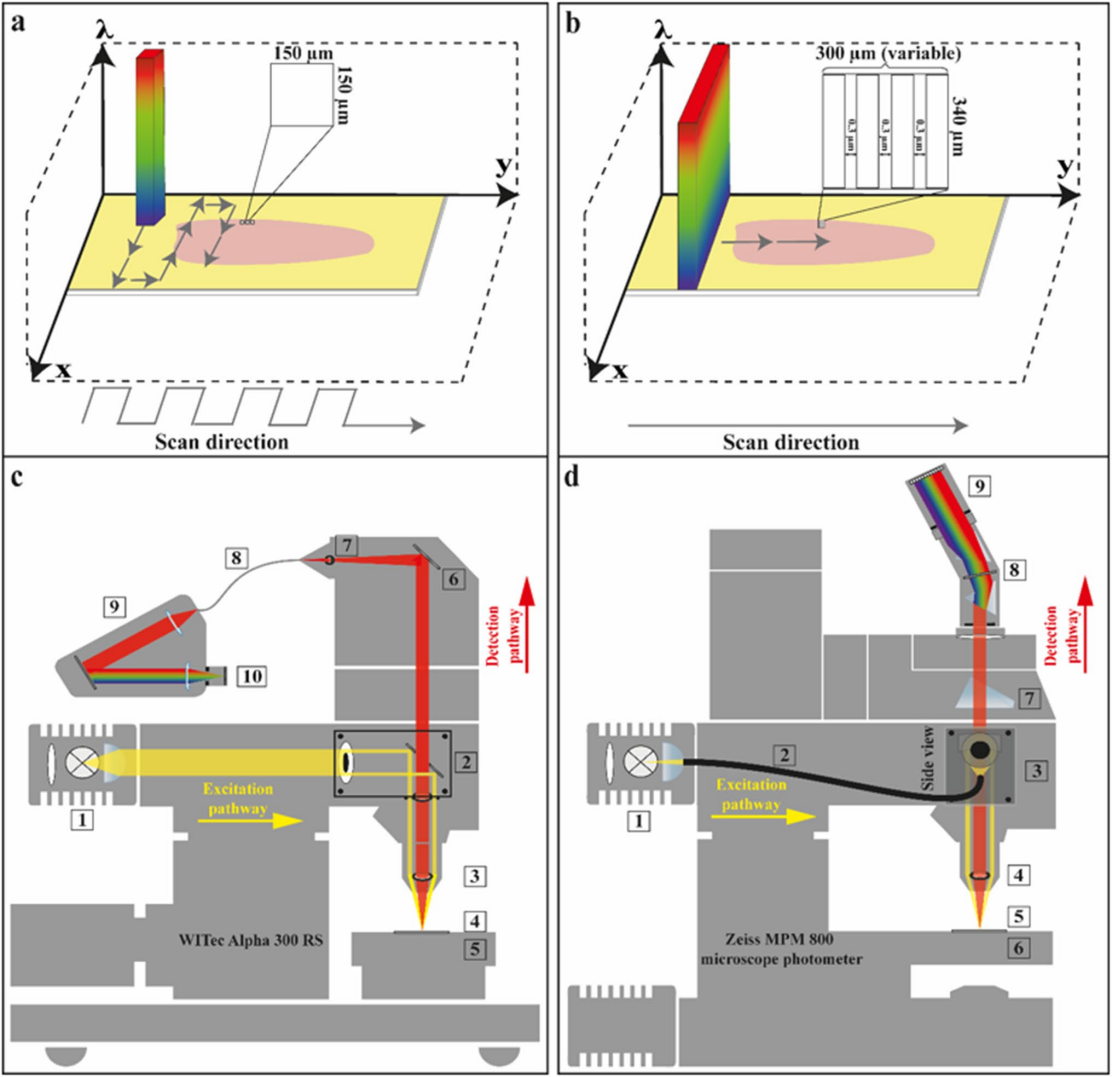
Comparison of Whiskbroom and Pushbroom principles (a, b) accomplished by the two instrumental setups (c, d). In Whiskbroom imaging (a), the sample is scanned point-by-point and a whole spectrum is recorded at each x, y-coordinate. The final spectral image derives from all single-point measurements combined. Each scanned point along the tongue was performed in a step-wise manner and is equivalent to a 150 µm × 150 µm scan size, as depicted by the zoom-in of one scan area. The small scan sizes illustrated on the mouse tongue should reveal the size proportion between scan area and tongue. In Pushbroom imaging (b), a complete line of pixels is measured simultaneously and full spectra are acquired in each pixel. The length of each scan in x-direction is fixed to 340 µm whereas the movement in y-direction is variable. Applied scan ranges in y-direction encompassed 180–300 µm. Ideal step sizes were predetermined to be 0.3 µm. One image consists of the scanned x- and y-direction. Whiskbroom imaging was performed by the WITec instrument (c) and Pushbroom imaging was executed by the MPM microscope photometer equipped with a Pushbroom detection system (d). WITec components are described by (c1–c10), c1: tungsten lamp, c2: darkfield module, c3: darkfield objective, c4: sample holder, c5: piezo scan table, c6: deflection mirror, c7: pinhole, c8: multimode optical fiber, c9: spectrometer, c10: CCD camera. MPM components are displayed by (d1–d9), d1: tungsten lamp, d2: optical fiber for white light transmission, d3: darkfield module (side view), d4: darkfield objective, d5: sample holder, d6: scan table, d7: prism, d8: spectrometer with optical elements, d9: imaging CCD camera

The principle of Whiskbroom and Pushbroom imaging is illustrated in Fig. 3a and b. In Whiskbroom imaging (Fig. 3a), the sample is scanned in a point-by-point manner acquiring a whole spectrum at each x, y-coordinate. In the end, the spectral image is obtained by combining all single-point measurements. In Pushbroom imaging (Fig. 3b), a complete line is measured at once while a spectrum at each pixel of the line is recorded. Pushbroom imaging therefore allows a much faster sample scanning compared to Whiskbroom imaging. In general, the two techniques are accompanied by varying but high spatial and spectral resolutions. Both imaging modes are applied for detecting ELS from tissue specimens within the scope of our study.

Whiskbroom images were recorded with a WITec Alpha 300 RS confocal system modified as described earlier [26] (Fig. 3c). The WITec instrument was equipped with a tungsten lamp (Osram, model HLX 64625), a DF module, and a 20 × DF objective (Zeiss, EC Epiplan Apochromat 20 × /0.6 HD DIC M27). A 100 µm-core diameter multimode fiber connects the optical output to the Acton SP2300i mirror-based spectrometer fitted with an Andor DU401 DD, 35 CCD camera (EMCCD, 16 Bit, 1024 × 127 pixel, 26 µm × 26 µm, operating temperature: − 60 °C). The spectrometer was centered at 700 nm using a 150 g/mm (BLZ = 800 nm) grating. A spectral range of 412–975 nm was thus measurable. The complete optical setup achieves a spectral resolution of 1.6 nm. Maximum scan areas encompass 150 × 150 µm limited by the range of the piezo table. Each spectrum was obtained with an integration time of 0.04 s. A total number 400 ELS spectra (20 × 20 spectra) was acquired in a scan area of 150 × 150 µm. The applied scan steps equal to 7.5 µm and the scan speed corresponds to 0.874 s/line. Spectralon® was used as reference material measured with the same acquisition parameters. The instrumental dark current was additionally determined as dark current spectrum. Following data acquisition, ELS images were normalized with Spectralon®, and dark current spectra were subtracted.

Additionally, an ELS hypercube was generated with a Zeiss MPM 800 microscope photometer implemented with a Pushbroom imaging system. The Pushbroom imager consisted of a spectrograph (Inno-Spec) and a CCD camera (QImaging, EXi Blue fluorescence microscopy camera, model: EXI-BLU-R-F-M-14-C) (Fig. 3d). Polychromatic light is generated by a tungsten lamp (Osram, model HLX 64625) and transferred via an optical fiber (core diameter 6.35 mm) onto the tissue sample. Elastically back-scattered light was collected by the above-described 20 × DF objective (Zeiss, EC Epiplan Apochromat 20⨯/0.6 HD DIC M27) and was finally recorded with the Pushbroom imager. The entrance slit dimension of the spectrometer corresponds to a width of 30 µm and a length of 14 mm. The spectral range encompasses 398–715 nm using a 600 g/mm grating. The area of light-sensitive pixels on the Pushbroom CCD chip contains 1392 × 1040 pixels whereby the first pixel number depicts the spectral axis (398–715 nm) and the second one represents the lateral axis. Each measured line-image exhibits a spatial width of 340 µm consisting of 1040 pixels. The lateral resolution is therefore 3 pixels/µm. Scan table increments were optimized to 0.3 µm. The number of line image scans depends on the actual tissue region and can vary between scans. The scan speed is 10 s/line. A spectral resolution of 1.2 nm can be defined for our customized setup. Comparable to the Whiskbroom data treatment, Spectralon® reference spectra as well as dark current spectra were acquired.

Corresponding excitation and detection light paths for the WITec Alpha 300 RS and the Zeiss MPM 800 microscope photometer are visualized in Fig. 3c and d. By implementing a DF illumination, the incident light laterally impinges on the specimen. Thus, only the diffuse ELS is detected whereas specular light is undetectable (Fig. 3c and d). Otherwise, the specular reflected light from the tissue superimposes the diffuse ELS carrying the information content and therefore hampers its detection. Due to the DF setup, no additional glare removal is necessary [36] and the diffuse ELS measurement of the sample is facilitated. Furthermore, we prepared our tissue samples on gold-coated objective slides in order to amplify the ELS of the specimen (Fig. 3a and b). Based on these adjustments, we intend to improve the overall ELS detection in order to achieve high-quality ELS data.

### Comparability of Whiskbroom and Pushbroom data by spatial averaging

In order to compare Whiskbroom and Pushbroom data in a set scan area, a spatial averaging of spectra must be performed. This is due to the different lateral resolutions of both methods. In our study, a scan area size of 75 × 75 µm yields 100 ELS spectra for Whiskbroom imaging, whereas Pushbroom imaging generates 50,625 ELS spectra in the same area. By averaging 100 spectra for Whiskbroom into one spectrum and 50,625 spectra for Pushbroom imaging into a second spectrum, comparability can be ensured. These averaged spectra for both methods thus represent the identical area size of 75 × 75 µm. Although DF imaging can achieve high lateral resolutions of 0.5–1 µm, the obtained information content is too detailed for our purpose. Therefore, we chose the above-mentioned area size of 75 × 75 µm.

For the Whiskbroom PCA-DA model, a total number of 48 EP, 28 GM, and 60 AT mean spectra were generated, as described above (100 spectra averaged to one spectrum for a defined area size of 75 × 75 µm). The Pushbroom model was created by 33 EP, 24 GM, and 44 AT mean spectra (50,625 spectra averaged to one spectrum for a defined area size of 75 × 75 µm). For prediction purposes, a test set of spectral data was additionally measured and averaged in the previously described manner. The Pushbroom prediction data consisted of 6 EP, 7 GM, and 22 AT average spectra. On the contrary, Whiskbroom data yielded 4 EP, 4 GM, and 16 AT average spectra.

### Data preprocessing and PCA-DA model development

For PCA-DA model formation and testing, the software The Unscrambler® X (Camo Software, Version: 10.5) was used. Spectra of both acquisition methods were preprocessed equally with the software. At first, ELS image spectra were displayed as absorption spectra (-log(R)). A spectral smoothing according to Moving Average with 47 segment points was applied. Next, a baseline offset correction was performed and followed by a gap derivation (1^st^ derivative, gap size: 15 pts.). All spectra were conclusively range-normalized.

A PCA for Whiskbroom and Pushbroom spectra was calculated with mean-centering using the NIPALS-algorithm and the Leverage correction method for validation purposes. A total number of four principal components (PC) was required to represent the spectral data. Model outliers were displayed by the influence plot illustrating the *F*-residuals vs. the Hotelling’s *T*^2^ statistic with a critical limit of 5%. Spectral outliers were manually verified and removed from the model if proven to be true. Outlier spectra mostly appeared to be noisy background spectra measured in tissue holes. In combination with the PCA, a DA was accomplished with a quadratic distance calculation using four PCs.

Several validation parameters like sensitivity, specificity, and precision were calculated for both PCA-DA models in accordance with the confusion matrix terminology. They enabled a characterization of the models and demonstrated their functionality. All three parameters were computed as following

$$Sensitivity=\frac{True\;Positives}{True\;Positives+False\;Negatives}$$

$$Specificity=\frac{True\;Negatives}{True\;Negatives+False\;Positives}$$

$$Precision=\frac{True\;Positives}{True\;Positives+False\;Positives}$$

Each validation parameter was weighted considering the number of ELS tissue spectra, which contribute to the model.

The graphical representation of spectral and statistical data is executed with OriginPro 2018G (OriginLab Corporation).

## Results

### Histology of murine tongue tissues

**Fig. 4:**
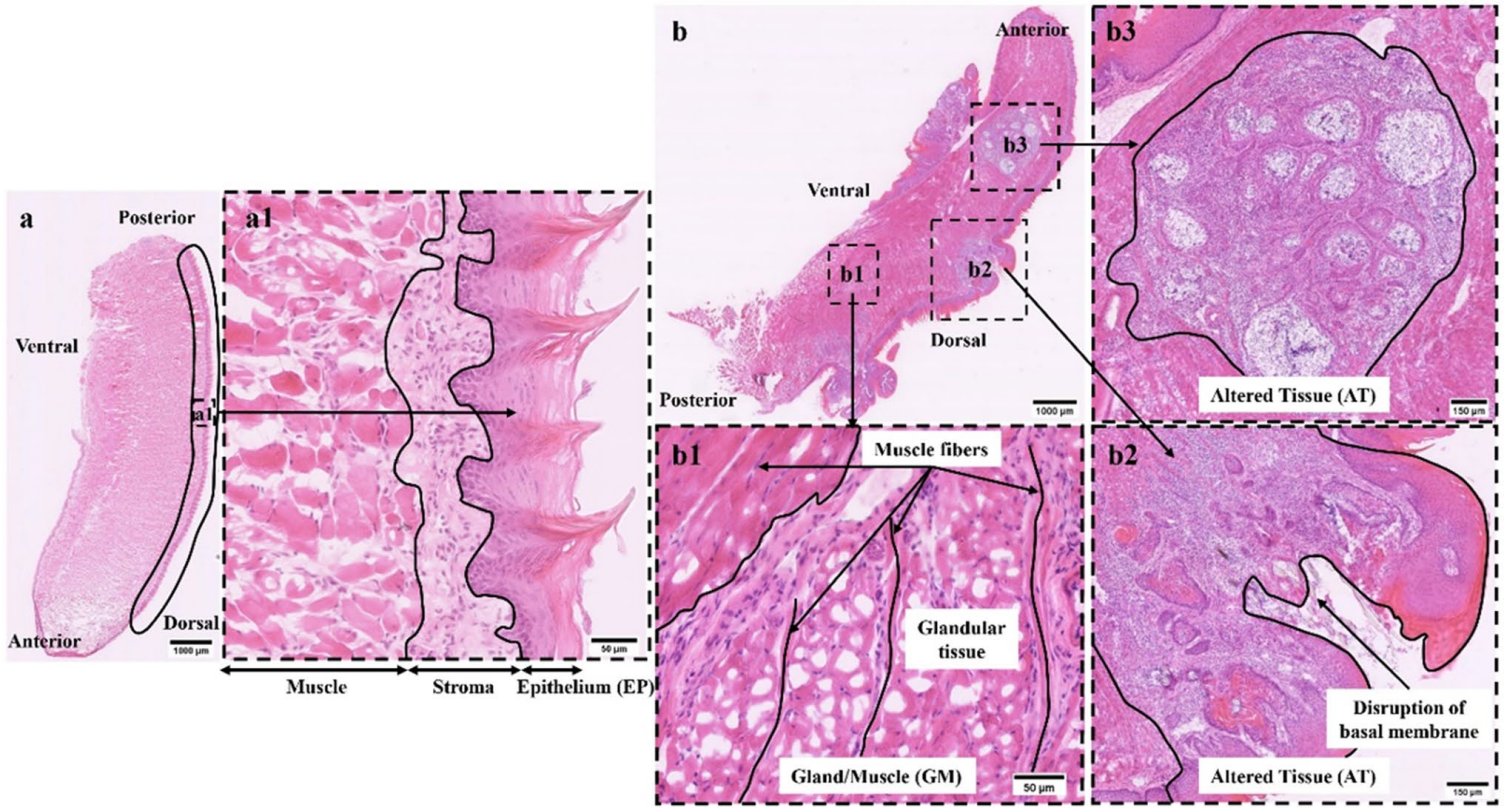
Histological description of representative tissue regions for epithelium (EP), gland/muscle (GM), and altered tissue (AT). A longitudinal tongue section of mouse a with its anatomical orientation is shown in a. Epithelium in dorsal direction is marked by the black frame (a). An enlarged view of the dorsal epithelium (a1, dotted line) points out its multilayered structure. The keratinized top layer merges into a metabolically active epithelium layer allying to the stroma. The metabolic active epithelium is separated from the stroma by the basement membrane. Within the uppermost layer, filiform papillae are embedded in a tip shape. Typical gland/muscle (b1, dotted line) and OSCC tissue areas (b2, b3, dotted line) are located in an overview tissue section of mouse C (b). Glandular tissue is mainly implemented into muscle tissue indicated by the vertically oriented muscle fibers (b1). OSCCs are visible in b2 and b3. OSCC undergoes epithelial-mesenchymal transition, invading subjacent tissues and interrupts the highly organized epithelium layer (b2). One OSCC branch additionally exhibits adenocarcinoma parts (b3)

The identification of different tissue types within a tongue tissue section requires a histological assessment, commonly performed using HE-staining. For our study, various tissue regions of longitudinal-cut murine tongue cross sections were HE-stained and histologically classified by our pathologist Marion Roeßler. Based on the pathologist’s evaluation, two distinct healthy tissue areas could be distinguished in all mice, from which epithelium and stroma could be assigned. Epithelial tissue defines the outermost layer of the tongue lining the entire organ from the ventral to the dorsal side (Fig. 4a). The epithelial tissue consists of several cell layers, which are a highly proliferative basal region, followed by a dense and metabolically highly active suprabasal layer and finally a keratinized layer on top that forms the filiform papillae of the dorsal tongue (Fig. 4a1, dashed area). Due to the layered structure of the epithelium, this tissue is highly heterogeneous from a cross-sectional point of view. The epithelium sits on a stromal region that consists of fibroblasts, immune cells, and small capillaries in conjunction with extracellular matrix. Adjacent to the epithelial stroma, a mixed-tissue of gland and muscle, the stroma, affiliates (Fig. 4a1). Muscle fibers run along the cross-sectional middle part of the tongue in a highly defined and structured manner (Fig. 4b1). In-between those muscle fibers, glandular tissue is embedded, but mainly located beneath the epithelial layer (Fig. 4b and b1). Carcinogen-treated mice additionally display morphological or cell-structural alterations of epithelial tissue with several tumor stages defined as hyperplasia, dysplasia, or SCC. An invasive SCC has been characterized in our mouse tongues originating from healthy epithelium after carcinogen treatment (Fig. 4b2, b3). Early epithelial changes include thickened epithelium and hyperkeratosis, followed by cellular and nuclear pleomorphism with abnormal cellular size and shape changes, nuclear hyperchromatism, and increased and abnormal mitotic figures. Moderate dysplasia presents loss of cell polarity, disordered maturation from basal to squamous cells, and increased cellular density. Invasive SCCs grow into underlying tissue areas by disrupting the basal membrane and loss of epithelial stratification (Fig. 4b2). In some specimens, SCCs also show invasion into gland and muscle layers (Fig. 4b). One SCC tissue segment could even be specified as a carcinoma variation with an adenocarcinoma part (Fig. 4b3) [37]. Representative areas of healthy and altered tissue regions were chosen for DF ELS imaging according to the pathologist’s evaluation.

### Whiskbroom PCA-DA model

**Fig. 5:**
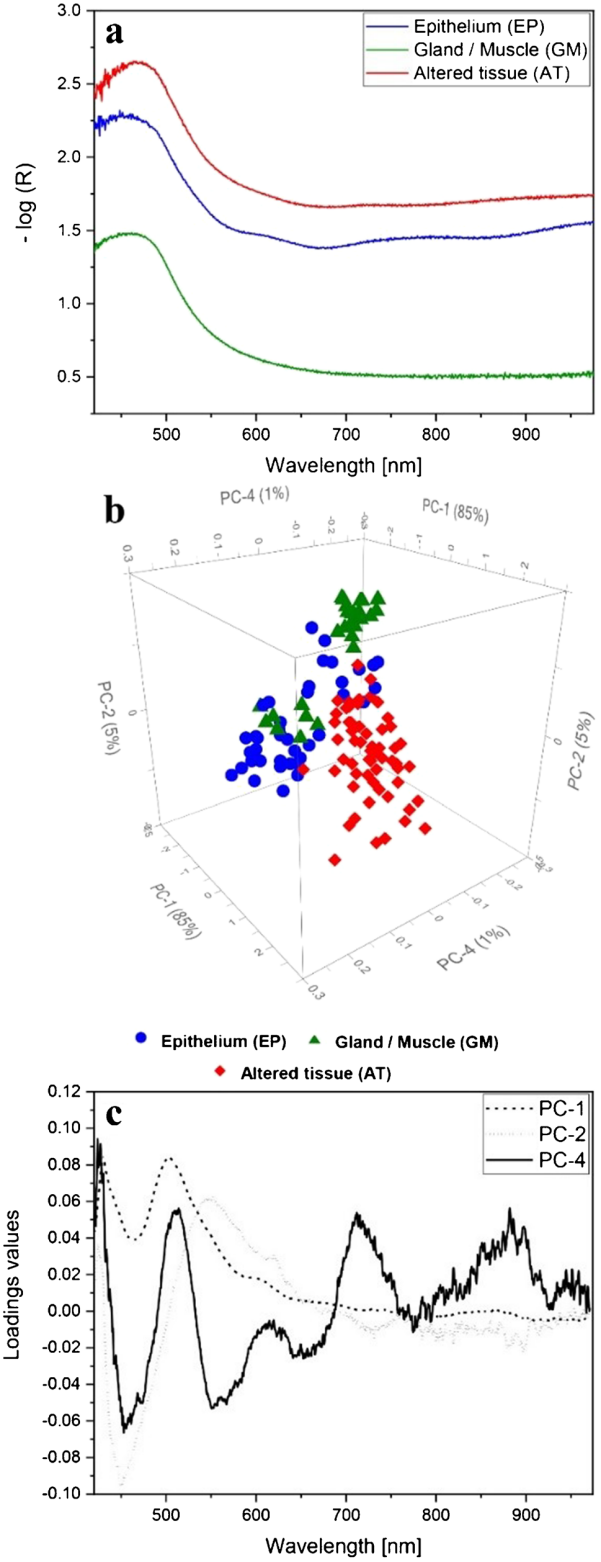
PCA model based on Whiskbroom data for the differentiation of EP (blue), GM (green), and AT (red). In a: exemplary ELS spectra of EP (blue), GM (green), and AT (red) tissue are depicted without any data preprocessing steps. They are displayed as absorption spectra (-log(R)). In b: the 3D scores plot of the PCA reveals the separation of GM, EP, and AT groups by PC1, PC2, and PC4 explaining 85%, 5%, and 1% of the overall variance. It shows the importance of a third PC to achieve a distinctly improved segregation. Only preprocessed ELS spectra are considered for the PCA calculation. Preprocessing steps encompass: moving average smoothing with 47 segment points, baseline offset correction, gap derivation (1^st^ derivative, gap size: 15 pts.), and range-normalization. In c: corresponding loadings plots for each PC of the calculated PCA. In Supplementary Material: the 2D scores plot of PC4 against PC3 as well as PC3 against PC2 with the related loadings plots are shown (Fig. S2)

All tissue type spectra were area-averaged, as described above, to a final number of 48 EP, 28 GM, and 60 AT mean spectra used for the PCA-DA model formation (“Materials and methods”). The used spectra originated from three different mice (mouse A–C). In Fig. 5a, three exemplary Whiskbroom ELS spectra of EP, GM, and AT without any data preprocessing steps are shown. The ELS spectra are dominated by a wave-like appearance superimposed by even finer scattering patterns [38]. These typical features are not always directly visible in ELS spectra since the diffuse scattering intensity is often weak and the spectral shape is dominated by the gold substrate (Fig. 5a). The y-axial intensity displacement among EP, GM, and AT spectra can mainly be ascribed to influences of different slice thicknesses (Fig. 5a). Thus, data preprocessing is mandatory to extract the hidden scattering information from the ELS spectra and to remove unwanted effects. The Whiskbroom PCA-DA calculation was only accomplished with preprocessed ELS spectra (“Materials and methods”). Exemplary preprocessed spectra are illustrated in Supplementary Material, Fig. S1a.

The resulting PCA for ELS Whiskbroom spectra is illustrated in Fig. 5b and c. The 3D scores plot shows PC1, PC2, and PC4 representing 85% for PC1, 5% for PC2, and 1% for PC4 of the total variance. All three PCs are required to achieve a separation of the investigated tissue types. Although partly overlapping, each tissue type forms one conglomeration. PC1 assigns mostly below-average score values to the spectral GM and EP clusters whereas the AT group is mainly characterized by above-average score values (Fig. 5b). In contrast to PC1, the impact of PC2 is less pronounced since it only affects a complete separation of the GM agglomeration from the AT group. It does not improve the segregation of the EP cluster. The EP group lies diagonally in-between GM and AT within the PC2 vs. PC1 plain of the scores plot (Fig. 5b). Overall, PC2 promotes a slanted differentiation of the three tissue-type clusters. Corresponding loadings plots of PC1 and PC2 reveal the greatest effects on the cluster formation in a wavelength region of 400–600 nm with two loadings maxima at 430 nm and 503 nm for PC1 and one major negative maximum at 540 nm for PC2 (Fig. 5c, dashed and dotted line). Above 600 nm, a poorly distinct ripple structure is visible for both loadings plots. The loadings curves demonstrate the effect of the spectral ELS signature on the tissue cluster separation. PC4 finally enables a significantly improved separation of all three tissue agglomerations (Fig. 5b). The main influence of PC4 is the differentiation of GM and EP spectra from one another and it more precisely defines and shapes the tissue clusters. Examination of the PC4 loadings plot shows a repeatedly occurring pattern partly overlaid by even smaller ripple elements, which refer to Mie scattering (Fig. 5c, solid line). The segregation of the tissue clusters is dominated by these effects.

Based on the PCA calculation, a DA was subsequently computed and combined to a final PCA-DA model that allows the classification of ELS spectra from unknown lingual tissue areas. The actual model was created by a training set of ELS spectra for EP, GM and AT and optimized with respect to best classification results. By applying the DA, a validation of the used quadratic distance algorithm with 4 PCs was performed and illustrated as a confusion matrix (s. Supplementary Material, Tab. S1). As part of the confusion matrix results, the training spectra themselves were predicted by the algorithm and afterwards grouped dependent on the prediction outcome. An overview of the training spectra prediction is summarized in Table 2. Except for the AT group, the DA algorithm correctly predicted all EP and GM training sets (Table 2). The overall model accuracy corresponds to 98% for the Whiskbroom data in total.

**Table 2:** Model-related quality parameters of the Whiskbroom PCA-DA. In addition to the total number of training spectra (column 2), their prediction results by the DA are listed as absolute numbers and in percentage terms (columns 3 and 4). The last columns (columns 5–8) represent the overall accuracy, sensitivity, specificity, and precision of the PCA-DA model

Tissue type	Total spectra in model	Correctly predicted model spectra	Proportion of correctly predicted model spectra [%]	Accuracy [%]	Sensitivity [%]	Specificity [%]	Precision [%]
Gland/muscle (GM)	28	28	100	98	98	99	98
Epithelium (EP)	41	41	100				
Altered tissue (AT)	58	56	97				

For a precise characterization of the PCA-DA, further model parameters such as sensitivity, specificity, and precision were determined to represent the model’s performance. The parameters were calculated from the confusion matrix (s. Supplementary Material, Tab. S1) and describe the model quality. Dependent on the amount of ELS training data for each tissue type, the training spectra contribute differently to the calculation of the respective model parameters. This contribution was considered in terms of different weightings for the tissue groups. A summary of the weighted sensitivity, specificity, and precision is presented in Table 2. The Whiskbroom model shows holistic sensitivity and precision values of 98% and even exhibits a specificity of 99%. These parameters demonstrate that the model was optimized as effectively as possible with the training set. Thus, reasonable classifications of unknown ELS spectra are expected.

### Pushbroom PCA-DA model

**Fig. 6:**
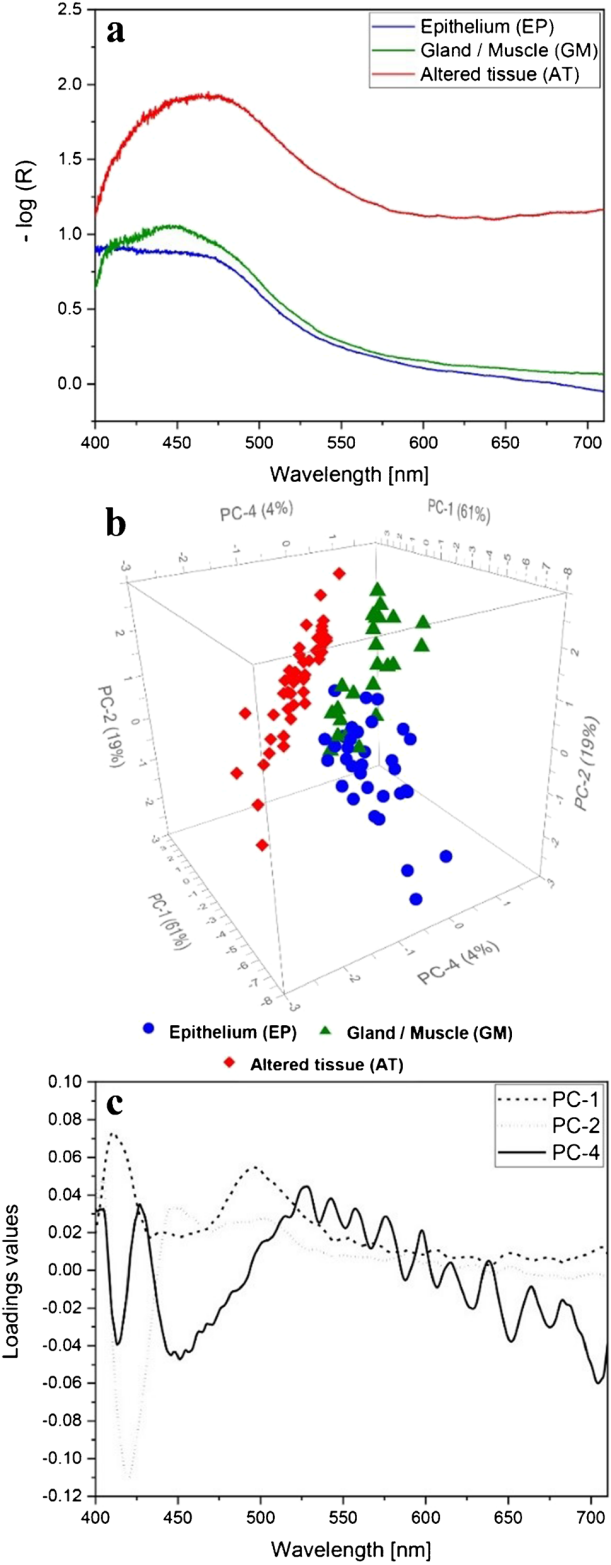
PCA model based on Pushbroom data for the differentiation of EP (blue), GM (green), and AT (red). In a: exemplary ELS spectra of EP (blue), GM (green), and AT (red) are depicted without any data preprocessing steps. They are displayed as absorption spectra (-log(R)). In b: the 3D scores plot of the PCA reveals the separation of GM, EP, and AT groups by PC1, PC2, and PC4 explaining 61%, 19%, and 4% of the overall variance. It shows the importance of a third PC to achieve a distinctly improved segregation. Only preprocessed ELS spectra are considered for the PCA calculation. Preprocessing steps encompass: moving average smoothing with 47 segment points, baseline offset correction, gap derivation (1^st^ derivative, gap size: 15 pts.), and range-normalization. In c: corresponding loadings plots for each PC of the calculated PCA. In Supplementary Material: the 2D scores plot of PC4 against PC3 as well as PC3 against PC2 with the related loadings plots are shown (Fig. S3)

For the Pushbroom PCA-DA model, 33 EP, 24 GM, and 44 AT spectra were area-averaged in the above-described manner (“Materials and methods”). All spectra were obtained from three different mice (mouse A–C). Exemplary Pushbroom spectra of each tissue type without any data preprocessing steps are illustrated in Fig. 6a. As mentioned above, ELS spectra show a typical sinusoidal shape overlaid by smaller scattering patterns [38] which is hardly visible in the Pushbroom spectra (Fig. 6a). Therefore, a data preprocessing is again necessary to gain the actual scattering information and remove measuring- or sample-ascribable effects, such as the y-axial intensity displacement due to varying slice thicknesses or the impact of the gold substrate on the overall spectral trend (Fig. 6A). The Pushbroom PCA-DA calculation was only accomplished with preprocessed ELS spectra (“Materials and methods”). Exemplary preprocessed spectra are illustrated in the Supplementary Material, Fig. S1b.

The PCA for the ELS Pushbroom spectra is displayed in Fig. 6b and c. Three PCs are required to achieve an almost complete segregation of the EP (blue), GM (green), and AT clusters (red). The 3D scores plot shows PC1, PC2, and PC4 representing 61% for PC1, 19% for PC2, and 1% for PC4 of the total variance. Due to PC1 and PC2, the tissue clusters are arranged around the scores plot center with only minor overlapping of all three groups. PC1 mainly separates the GM and EP tissue clusters from the AT conglomeration. Since GM and EP are mostly defined by below-average score values and AT by above-average ones, all groups are organized in the described manner. PC2, however, primarily enables a segregation of the EP group from the GM and AT clusters with a partial overlay of the conglomerations. The related loadings plots for PC1 (Fig. 6c, dashed line) and PC2 (Fig. 6c, dotted line) explain the arrangements of the three tissue groups in the scores plot based on dominating spectral effects. Within the loadings plot for PC1 (Fig. 6c, dashed line), two spectral maxima at 411 nm and 495 nm are observable. The loadings plot for PC2, however, shows one main negative peak at 420 nm. For both, a periodic spectral course is implied in a higher wavelength range of 550–700 nm. PC4 is essential to achieve an almost complete differentiation of the EP, GM and AT aggregation (Fig. 6b). The scores plot (Fig. 6b) demonstrates that PC4 not only accomplishes a total segregation of the AT cluster from EP and GM, but also further separates the EP group from the GM one. Nevertheless, a slight overlapping between the EP and GM clusters remains. The examination of the PC4 loadings plot (Fig. 6c, solid line) reveals negative maxima at 411 nm and 450 nm as well as a distinct spectral ripple pattern dominating above 530 nm which refer to Mie scattering (Fig. 6c, solid line).

Subsequently, a DA was calculated, as already described for the Whiskbroom model. By assigning the ELS training spectra with the used DA algorithm, the model performance was verified. The performance results were summarized in a confusion matrix (s. Supplementary Material, Tab. S2). As the majority of training spectra were correctly matched by the model, an overall accuracy of 98% was achieved (Table 2). All investigated AT spectra were entirely dedicated to AT, whereas 32 EP and 23 GM spectra out of 33 EPs and 24 GMs were also attributed to EP and GM, respectively.

Identical model parameters were also calculated to characterize the model more accurately (Table 3). They indicate the model’s applicability for classification purposes. All parameters were again calculated as weighted values that consider the different quantities of EP, GM, and AT spectra in the training set (Table 3). Results for the weighted accuracy, sensitivity, specificity, and precision were comparable to the Whiskbroom model.

**Table 3:** Model-related quality parameters of the Pushbroom PCA-DA. In addition to the total number of ELS training spectra (column 2), the classification results of the training set were listed as absolute numbers and in percentage terms (columns 3 and 4). The last columns (columns 5–8) represent the overall accuracy, sensitivity, specificity, and precision of the PCA-DA

Tissue type	Total spectra in model	Correctly predicted model spectra	Proportion of correctly predicted model spectra [%]	Accuracy [%]	Sensitivity [%]	Specificity [%]	Precision [%]
Gland/muscle (GM)	24	23	96	98	98	99	98
Epithelium (EP)	33	32	97				
Altered tissue (AT)	44	44	100				

### Classification of tissue types by PCA-DA models

**Fig. 7:**
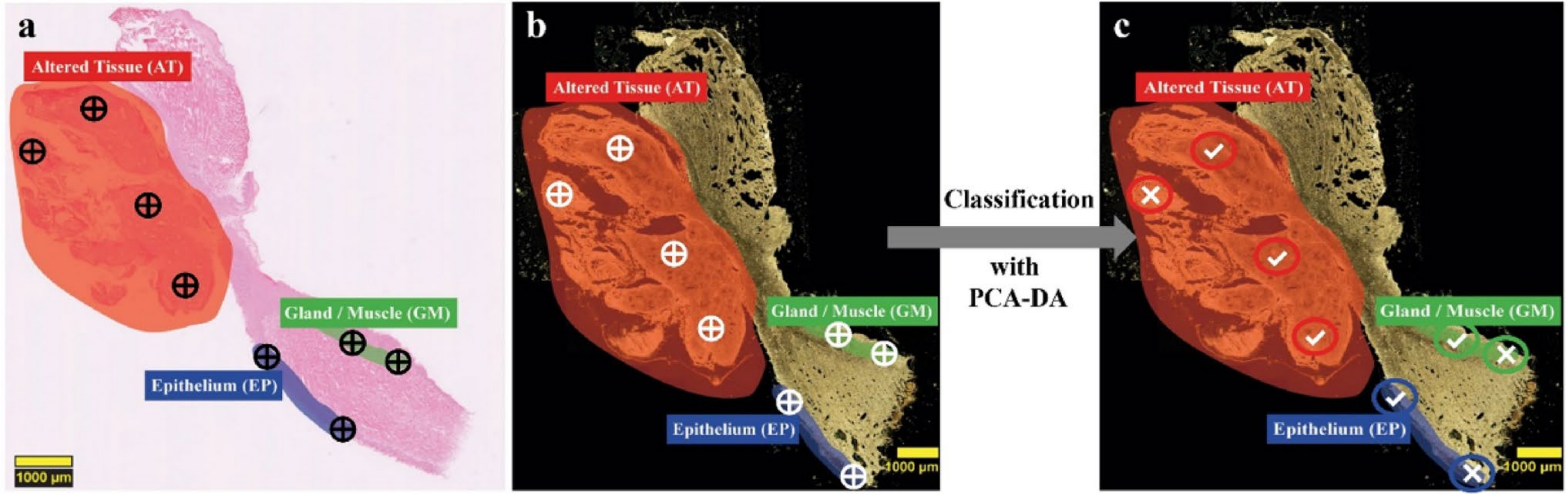
Classification principle of the PCA-DA models. In a: a HE-based identification of different tissue types from a to-predict tissue section. The crosshairs in a indicate the tissue spots, which need to be located in b for ELS spectra measurements. In b: localization of the same tissue regions and spots on corresponding tissue sections used for ELS data acquisition. According to this localization, ELS test spectra for the different tissue types were measured at each spot (white crosshairs) and were afterwards classified by the PCA-DA models. In c: as implied by the white crosses or checkmarks, the prediction of the ELS test spectra by the model either coincides with the HE identification (checkmark) or differs from it (cross)

Our classification approach is visualized in Fig. 7. At first, a histopathological identification of all three tissue types by HE-stained tissue sections had to be performed in order to localize the tissues on the ELS-ready tissue sections (Fig. 7a and b). Based on this tissue assignment, ELS test spectra of the tissue types were acquired and finally classified by the PCA-DA model (Fig. 7b, white crosshairs). To validate whether the classification of the model is true, the classification results were compared with the HE diagnosis of the exact same tissue regions. As indicated by the white crosses or checkmarks (Fig. 7c), the prediction by the model either conforms to the HE-identification (checkmark) or differed from it (cross). The corresponding prediction results are summarized in Table 4.

**Table 4:**
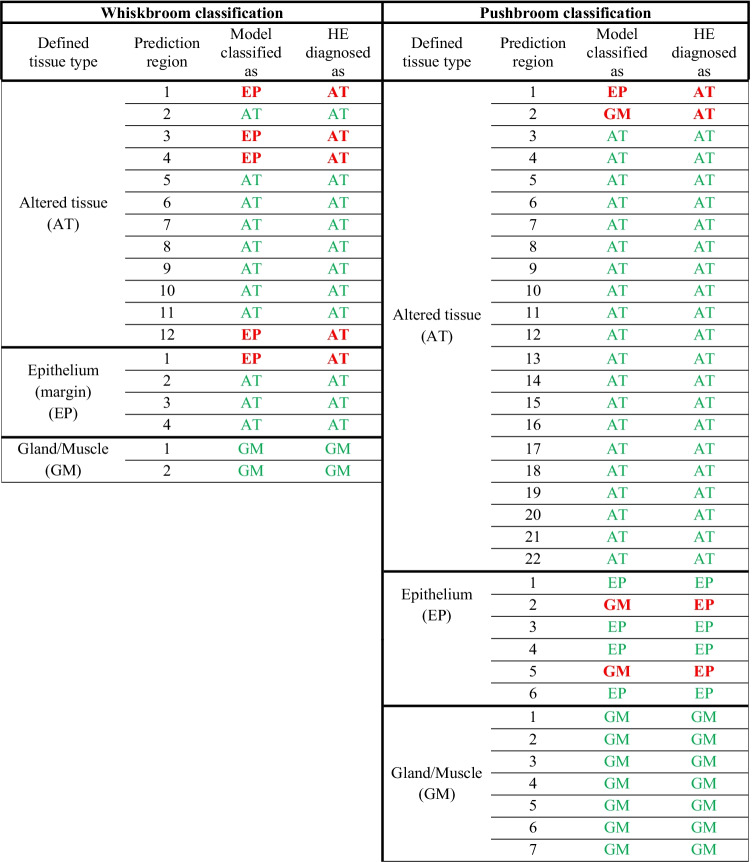
Summary of the PCA-DA model classification results and HE-diagnosed conclusions. ELS test spectra of different tissue regions for AT, EP, and GM were classified by both models and HE-evaluated by a blinded pathologist. Epithelium regions (1*–*4) of the Whiskbroom model adjoin to one of the AT areas and are thus declared as EP margin. If the model classification and the HE diagnosis coincide, they are marked in green. Otherwise, they are highlighted in red and bold

For both PCA-DA models, the test set of EP, GM, and AT spectra was obtained as described earlier (“Materials and methods”). Since the classification models were created by ELS training spectra of three different mouse samples (mouse A–C), the tissue of a fourth mouse (mouse D) was considered for testing the AT classification. The additional mouse sample should emphasize that the model can correctly assign unknown AT spectra of a completely new tissue sample. Furthermore, unknown EP and GM tissue regions of mouse samples A, B, and C were also examined with the PCA-DA models.

A classification of 12 different AT regions of mouse D, four EP regions, and two GM areas of mouse C was performed by the Whiskbroom model. A test set of 6 different EP spectra of mouse A, 7 GM spectra of mouse B, and 22 AT spectra of mouse D, however, was used to verify the classification abilities of the Pushbroom model. A summary of the model classification results and the alignment with the HE diagnosis is shown in Table 4. Since the PCA-DA models were mainly developed for the AT recognition in tongues, mostly AT tissues were included in the testing set. Overall, our classification procedure reveals the true prediction capability of our models.

## Discussion

In this proof-of-concept study, we compare Whiskbroom and Pushbroom DF ELSS imaging and apply both to a HNSCC mouse trial. ELS image data of murine lingual cross sections were acquired and subsequently pre-processed to eliminate recording-related influences. Afterwards, the ELS data were subjected to a PCA to allow a data reduction and extraction of the important tissues scattering information. By combining the PCA-structured ELS data with a DA, a Whiskbroom and Pushbroom PCA-DA model was formed. These models were used to classify unknown mouse tissue spectra and to distinguish between different tongue tissue types. A verification of the PCA-DA models was performed with an external set of ELS test spectra. Identical tissue regions were histologically evaluated and compared to the models’ classification results for validation purposes. This comparison finally allows determining if one of the PCA-DA models is more suitable to correctly identify the investigated tissue state. To our knowledge, the comparison of two different DF ELSS HSI detection principles in terms of tissue identification was conducted for the first time. So far, HSI only coupled with other spectral techniques was applied for the differentiation of healthy and cancerous tissue, such as Raman [39], fluorescence [40], or FT-IR imaging [25].

The application of a PCA is mandatory for the enormous amount of data recorded by Whiskbroom and Pushbroom imaging. The PCA is an objective and unbiased analyzing tool [41], which helps to structure the ELS data matrix. Suitable data preprocessing removes sample- and acquisition-associated effects, such as substrate background or section thickness. Due to the effects of PCA and data preprocessing, the main spectral influences for the tissue differentiation can be determined [42]. This is very important because the interpretation of ELS spectra is a demanding task as the spectral bands are not directly linked to a chemical group/vibration. The ELS patterns originate from various tissue constituents with changing refractive index, size and shape and are complicated to assign to one specific tissue component.

### Interpretation of Whiskbroom and Pushbroom PCA-DA models

A combined interpretation of cluster locations within the scores plot and the corresponding loadings plots allows a deduction of spectral influences that cause the tissue group separation. The tissue clusters in the Pushbroom PCA seem to be slightly better separated from one another with an overall less pronounced overlay compared to the Whiskbroom PCA (Fig. 5b, Fig. 6b). Analyzing the related loadings plots of PC1 and PC2 for both PCAs (Fig. 5c, Fig. 6c) reveals great effects within a wavelength range of 400–600 nm. Since PC1 of both PCAs separates the two healthy tissue groups from the tumor cluster, the impact of this wavelength region could be ascribed to changes in the cell nuclei of the tissues [43]. Nuclei in tumorous tissues are often enlarged and vary in size and shape compared to healthy nuclei [44]. Within the same wavelength range, the PC2 loadings plot shows a negative maximum at 450 nm for the Whiskbroom and at 430 nm for the Pushbroom PCA (Fig. 5c, Fig. 6c). In this case, this effect is mostly responsible for the cluster order along the PC2 axis. Based on the Whiskbroom cluster order, the below-average PC2 loadings maximum could give a hint to the structural organization of the three tissues. Since AT arises from intact EP, some tissue areas within the cancer region can still be healthy and thus might have the typical epithelium structure. This would explain the higher overlapping degree of the AT and EP group regarding PC2. The GM, however, is built up in a completely different manner in comparison to the EP or AT and consequently forms the most separated cluster. A small overlay of EP and GM groups according to PC2 can be explained by the close proximity of both tissues. For the Pushbroom PC2, however, the tissue group order is different. Although histologically identical tissue regions were chosen, the tissue structure and heterogeneity of the selected areas can differ compared to the Whiskbroom model and thus result in a varying cluster order. Above 600 nm, a sinusoidal curve progression is additionally indicated in both PC1 and PC2 loadings plots (Fig. 5c, Fig. 6c). These curve shapes are presumed to result from the superposition of many scattering events. Such patterns are amplified by the underlying gold substrate because of the gold’s pronounced reflectivity above 600 nm [45]. The application of PC4 causes an even more improved tissue cluster segregation for the Whiskbroom and Pushbroom PCA (Fig. 5b, Fig. 6b). This improvement can directly be attributed to the periodic structure that dominates throughout the entire spectral range in the PC4 loadings plots (Fig. 5c, Fig. 6c). Again, the periodic shape is expected to correlate with the scattering events related to cellular changes in the tissues [27, 38]. Alterations in cellular or subcellular units affect the sophisticated tissue structure and thus its morphology and texture [46, 47]. Differences in pattern frequency are observable in both PC4 loadings plots. The Whiskbroom PC4 loading reveals a long-wave sinusoidal shape superimposed by small ripple patterns whereas the Pushbroom loadings plot of PC4 is characterized by a ripple pattern of much higher frequency (Fig. 5c, Fig. 7c). A possible explanation can be deduced from the detection variations of both imaging setups. Since the Whiskbroom setup is a confocal scanning system, the scattering detection just takes place in the confocal volume and is thus very sensitive. Therefore, scattering of small tissue particles in addition to larger ones is accurately detectable influencing the observed pattern for the Whiskbroom PC4 loading. The high-frequency curve shape of the Pushbroom PC4 loading, however, might be correlated to the much larger detection spot of the Pushbroom setup. Therefore, no distinction of different tissue microstructures is possible and thus results in an overall scattering impression of higher frequency. Ninety-eight percent of all Whiskbroom and Pushbroom model-included spectra were accurately attributed to their corresponding tissue group (Tables 2 and 3). Additional model parameters confirmed the good performance of both models (Tables 2 and 3). Other studies could show comparable sensitivity values of 91% for detecting gastric tumors [48] and an accuracy of 88% in a colorectal ex vivo study [49]. By using a PCA-DA approach, a good tissue group formation could be achieved. The high degree of similarities between our models demonstrates the great robustness of DF ELSS imaging.

### Validation of PCA-DA models with HE-staining

A test set of ELS spectra was predicted by both PCA-DA models and identified either as EP, GM, or AT. The prediction outcome is compared with the HE diagnosis of identical tissue regions and verified whether the results match or not. For the Whiskbroom model, twelve tissue areas are expected to be AT. Eight of these regions were assigned to the AT cluster whereas the remaining four were diagnosed as EP tissue (Table 4). One explanation for this discrepancy in prediction is the tumor heterogeneity. Tumorous tissue cannot only consist of different tumor subpopulations, but also of histologically healthy areas [35, 50, 51]. Another plausible explanation could be that HNSCC tumors can exhibit variable levels of cellular differentiation. The same tumor can have regions of lower differentiation, possibly recognized as AT, whereas other, not healthy, regions are better differentiated and therefore classified as EP. A partial identification of the prediction regions as EP is thus possible. The corresponding HE diagnosis illustrates an overall AT impression and thus mostly coincide with the Whiskbroom results. Comparable outcomes were achieved by the Pushbroom model, which was able to assign 20 prediction regions out of 22 as AT tissue (Table 4). Only two areas were classified as EP and GM. The HE investigation confirmed that the tissue is modified. The two wrongly identified Pushbroom regions are once more explainable by healthy regions within an otherwise tumorous tissue [35, 52]. Additionally, two sets of EP spectra were tested against the Whiskbroom and Pushbroom models. The Whiskbroom PCA-DA was verified with four potential EP areas adjacent to an HE-identified carcinoma region (Table 4). These EP areas were chosen to investigate whether the tissue has already morphologically changed or is still intact. As a result, the model classified three of four EP regions as AT and only one was identified as epithelial tissue (Table 4). By close examination of the associated HE areas, all regions were categorized as AT. This comparison reveals how sensitive the ELS-based Whiskbroom model is in terms of detecting small morphological changes. Various EP regions were also assigned by the Pushbroom model. Four of six EP areas were attributed to the EP group and two of them were identified as GM (Table 4). The HE examination, however, revealed a distinct classification of all six test regions as EP. Both failing model predictions can be explained by the close vicinity of the GM tissue to the EP. For the GM prediction, two different testing regions were defined for the Whiskbroom and seven GM areas were chosen for the Pushbroom model (Table 4). Since the GM classification only plays a minor role, fewer ELS spectra were tested. Both GM regions were correctly allocated by the Whiskbroom PCA-DA, which also matches with the HE-evaluation (Table 4). Comparable results were achieved with the Pushbroom model and were additionally approved by the HE analysis. Here, seven out of seven test regions were identified as GM (Table 4). A high conformity with HE diagnosis was reached and thus the Whiskbroom and Pushbroom model predictions were proven reliable. The comparison of a statistical-based prediction model with the histopathology as the gold standard is a common approach and was also accomplished by others [53].

### Comparison of Whiskbroom and Pushbroom PCA-DA models

Both imaging models demonstrated good predictive skills, which are in high accordance with the HE diagnosis. The model structures of the Whiskbroom and Pushbroom PCA-DA models are almost identical in terms of number and types of PCs. Besides, a great similarity was also depicted for all three loadings plots of both models (Fig. 5c, Fig. 6c). Although the acquisition principles and resolutions differ between Whiskbroom and Pushbroom imaging, comparable statistical models could be formed with almost identical values for accuracy, sensitivity, specificity, and precision (Table 2, Table 3). Based on the prediction results, the Pushbroom model seemingly achieved an overall better classification than the Whiskbroom model for all investigated tissue types (Table 4). In this context, some aspects need to be considered. First, the overall number of ELS test spectra was higher for the Pushbroom prediction in comparison to the Whiskbroom one. Therefore, one or two misclassifications by the Whiskbroom model have a significant higher impact on the relative prediction outcome than for the Pushbroom model with a greater testing population. This creates the impression of a better Pushbroom prediction prognosis compared to the Whiskbroom one and favors this model in terms of its prediction capability. Considering identical testing populations, the correct classification ability might change between both models. For this reason, a comparison of the model’s prediction is difficult. Fortunately, a great amount of correctly predicted spectra and a small number of false classifications for both models occurred. Nevertheless, the differences of false prediction results between the models are minor and thus a clear capability preference for one or the other model is challenging to define. Second, the test regions of the EP margin turned out to be mostly AT as was confirmed by the Whiskbroom model and a detailed HE-diagnosis (Table 4). In further experiments, explicit EP regions need to be identified and examined by Whiskbroom imaging in order to validate whether the EP identification is fully possible. Still, the Whiskbroom results are reliable. The Pushbroom model testing, however, is thus more representative. Nevertheless, an enlarged testing of unknown tissue areas would be necessary to verify and confirm that the Pushbroom PCA-DA is the better model for the HNSCC application. An extension of the ELS training set for both models should also be realized to further advance their statistical validity. Additional improvements will encompass an expansion of the mouse model with a much higher amount of different HN tissue samples. Therefore, tissue heterogeneity will become less prominent from a statistical point of view.

For assessing whether the Whiskbroom or Pushbroom method is more suitable for the tissue classification, the different spatial and optical resolutions are considered. The applied spatial resolution for Whiskbroom imaging is equivalent to 7.5 µm. Using this resolution in combination with a confocal setup, no spatial information is mixed and an overview image of the investigated tissue extract is generated. Although higher spatial resolutions would have been possible with the instrumental setup, the detected spatial information content was sufficient to create an accurate Whiskbroom PCA-DA model. Based on this model, a high number of correct tissue assignments was achievable (Table 4). However, a high spatial resolution would be necessary to detect the small tissue-related differences in a single-point measurement fashion. This is particularly important for the recognition of tissue margins. Future experiments need to reveal whether the chosen spatial resolution is suitable for a potential tissue-border identification or if an adaption in resolution is required. For this study, most tissue classifications were performed with pathologically distinct tissue regions. Within this context, we thus demonstrated that the used Whiskbroom model is applicable to clearly classify these tissue regions, and our scanned images generated the spectral information necessary for this purpose. In order to resolve the spectral scattering patterns of each image, the high spectral resolution of 1.6 nm for the Whiskbroom setup is essential. The combination of this spectral resolution and the applied data preprocessing enabled the visualization and extraction of the Mie patterns with superimposed small ripples, as visualized by the loadings plots (Fig. 5c). Within the context of data preprocessing, a relatively high degree of spectral smoothing and a spatial averaging were applied. Both measures eliminated noise signals, but simultaneously maintained the superimposed small ripple patterns (Fig. 5c). Differences in tissue-section thicknesses were removed by a data normalization and are thus not represented by the scattering patterns (Fig. 5c). In addition to the spatial and spectral resolution of the Whiskbroom imaging, the chosen spectral range of 412–975 nm might also play an important role in tissue classification. In this spectral region, the 3^rd^ overtone of the NIR region is present and influences not only the model’s tissue cluster formation (Fig. 5b), but also the classification outcome of the tissue predictions. We ascertain the NIR region to be substantial for the PCA tissue cluster separation and also for the good classification results by the Whiskbroom model. Certainly, this also results from the high photon penetration in this spectral range. Compared to Whiskbroom imaging, the Pushbroom imager reveals a similar spectral resolution of 1.2 nm, but higher spatial resolutions in x- and y-direction. In x-direction, the resolution is diffraction-limited whereas in y-direction it is equivalent to 1.5 µm. The resolution in x-direction depends on the number of pixels along the axis, the microscope magnification power (20 ×), and the resolution limit of the optics. In y-direction, the spatial resolution is additionally influenced by the experimentally defined step size of 0.3 µm. The combination of spatial resolution and step size in y-direction results in a scanning overlap of 1.2 µm and thus five scanning steps cover identical spectral information. From a spatial perspective, Pushbroom images thus contain a much higher information content in comparison to Whiskbroom images. Each tissue section within the Pushbroom scan range is measured and small tissue differences can be gathered. Using this data, a robust Pushbroom PCA-DA model was formed and a high level of correct tissue predictions was accomplished (Table 4). We assume the high spatial resolutions to be much more appropriate for tissue-margin identification than the current Whiskbroom spatial resolution. In prospective experiments, the effect of the Pushbroom’s spatial resolutions in terms of border recognition also needs to be studied. So far, the applied classifications only included border-free tissue regions which were successfully predicted by the Pushbroom model. Therefore, the overrepresentation of identical spectral information due to the scanning overlap does not seem to negatively affect the classification outcome, as most prediction areas were correctly assigned by the model. Nevertheless, the spatial resolution in y-direction as well as the step size needs to be adjusted in a way that all the spatial information is covered and no scan time is wasted on identical spots. Additionally, the impact on the scan direction of the tissue (east to west, north to south, and vice versa) needs to be investigated. In combination with the overrepresentation of identical spectra, the earlier described data preprocessing (spectral smoothing and spatial averaging) might have caused in this case a loss of spectral information. Due to the additional spatial averaging, smaller Mie patterns might have been eliminated and the overall shape is much smoother in comparison to the Whiskbroom patterns, illustrated by the loadings (Fig. 6c). Still, the obtained spectral Mie patterns are representative enough to allow a distinct PCA clustering and a high level of correctly assigned tissue classifications (Fig. 6b, Fig. 6c, Table 4). One positive effect of the spectral overrepresentation might be an increasing robustness of the Pushbroom PCA-DA model which might also be reflected by the classification results. Compared to the Whiskbroom imaging, the spectral range of the Pushbroom imaging encompassed 398–715 nm and thus the 3^rd^ overtone of the NIR region is not considered. This is a drawback of our specific Pushbroom imager since the NIR region has an important impact on the tissue cluster formation and classification results, as stated earlier for the Whiskbroom model. We assume that the large statistics of the Pushbroom model compensates for the missing spectral impact of the NIR region and thus generates an even better model performance and classification ability compared to the Whiskbroom model.

Considering all the previously mentioned arguments, we assess the Pushbroom imaging to be more suitable for the detection and classification of our mouse tissue samples in comparison to Whiskbroom imaging. The authors point out that Pushbroom imaging can only be better suitable for a specific application than Whiskbroom imaging if the scanning overlap and spectral overrepresentation is optimized with an appropriate setup. Consequently, Pushbroom imaging as a fast, easy-applicable, and low-cost instrument might be applicable as a prospective diagnostic tool in a clinical daily routine.

## Conclusion

In conclusion, we successfully adapted two spectroscopic setups with a DF modulation to improve the detection of ELS and enabled the application of DF ELSS as imaging modality. One of the imaging systems was based on a Whiskbroom point-by-point scanning whereas the other was equipped with a Pushbroom imager to record ELS spectra in two different manners. Both imaging methods were employed on a HNSCC mouse model in a proof-of-concept study and generated a valuable ELS spectra set of different lingual tissue types. The ELS data were treated with several pre-processing steps to eliminate confounding factors of the sample or during the measurements. By assessing the treated ELS data with a PCA and a subsequent DA, we investigated whether DF ELSS imaging is capable of discriminating different tissue types and if either Whiskbroom or Pushbroom detection might be more suitable for this purpose. Our results show that DF ELSS is a very sensitive imaging technique able to distinguish between EP, GM, and AT based on their characteristic ELS pattern. PCAs for Whiskbroom and Pushbroom imaging revealed a distinct separation and thus differentiation of the three tissue type groups, although the Pushbroom PCA achieved a better cluster segregation. ELS is directly linked to the tissue’s morphology and thus the PCA separation is based on morphological changes between the tissues. Using both detection principles, accurate PCA-DA models were generated with which model-unknown ELS spectra were predicted and matched with the corresponding HE-staining. These results demonstrated that Whiskbroom and Pushbroom predictions are largely consistent with the histopathological evaluation. Nevertheless, we define the Pushbroom method to be more suitable for these samples with their specific absorption and scattering properties investigated in this study. Although the main goal of our study was the comparison of two DF ELSS imaging techniques and their suitability in HNSCC differentiation, the possible application of a Pushbroom setup as a non-destructive, cheap, and high-throughput technique in a clinical daily routine should be emphasized.

## Supplementary Information

Below is the link to the electronic supplementary material.

Supplementary file1 (PDF 721 KB)

## Data Availability

The data sets generated and/or analyzed during the current study are not publicly available since they are part of ongoing PhD theses. However, the data sets can be accessible on reasonable request from the corresponding author.
