# Supplementary material for: Comparison of Whiskbroom and Pushbroom darkfield elastic light scattering spectroscopic imaging for head and neck cancer identification in a mouse model
Source: Anal Bioanal Chem. 2021 Nov 19;413(30):7363–83. doi: 10.1007/s00216-021-03726-5 (PMC8626402; doi:10.1007/s00216-021-03726-5)
Supplement: Supplementary file 1 — Supplementary file1 (PDF 721 KB) [file 216_2021_3726_MOESM1_ESM.pdf]

Comparison of Whiskbroom and Pushbroom darkfield elastic light scattering spectroscopic imaging for head and neck cancer identification in a mouse model

Miriam C. Bassler[1,2], Mona Stefanakis[1,2], Inês Sequeira[3], Edwin Ostertag[1], Alexandra Wagner[1,2], Jörg W. Bartsch[4], Marion Roeßler[5], Robert Mandic[6], Eike F. Reddmann[1], Anita Lorenz[1], Karsten Rebner[1], *Marc Brecht[1,2]

*Corresponding author e-mail: marc.brecht@reutlingen-university.de

[1]*Process Analysis and Technology (PA&T) Reutlingen University, Alteburgstr. 150, 72762 Reutlingen, Germany*

[2]*Institute of Physical and Theoretical Chemistry University of Tübingen, Auf der Morgenstelle 18, 72076 Tübingen, Germany*

[3]*Institute of Dentistry, Barts and the London School of Medicine and Dentistry, Queen Mary University of London, London, UK*

[4]*Department of Neurosurgery,* [5]*Department of Pathology,* [6]*Department of Otorhinolaryngology, Philipps University Marburg, Baldingerstraße, 35033 Marburg, Germany*

Keywords

Mie elastic light scattering spectroscopy, chemometrics / statistics, clinical / biomedical analysis, head and neck cancer, mouse tumor model, microspectroscopy

Supplementary Material

Data preprocessing is essential to extract the hidden ELS information from the obtained image spectra for Whiskbroom and Pushbroom imaging. Several preprocessing steps were performed in order to allow a tissue differentiation based on ELS from the lingual tissue specimens: moving average smoothing with 47 segment points, baseline offset correction, gap derivation (1st derivative, gap size: 15 pts.) and range-normalization. Exemplary preprocessing ELS spectra for EP, GM and AT, acquired by both imaging methods, are shown in Fig. S1.

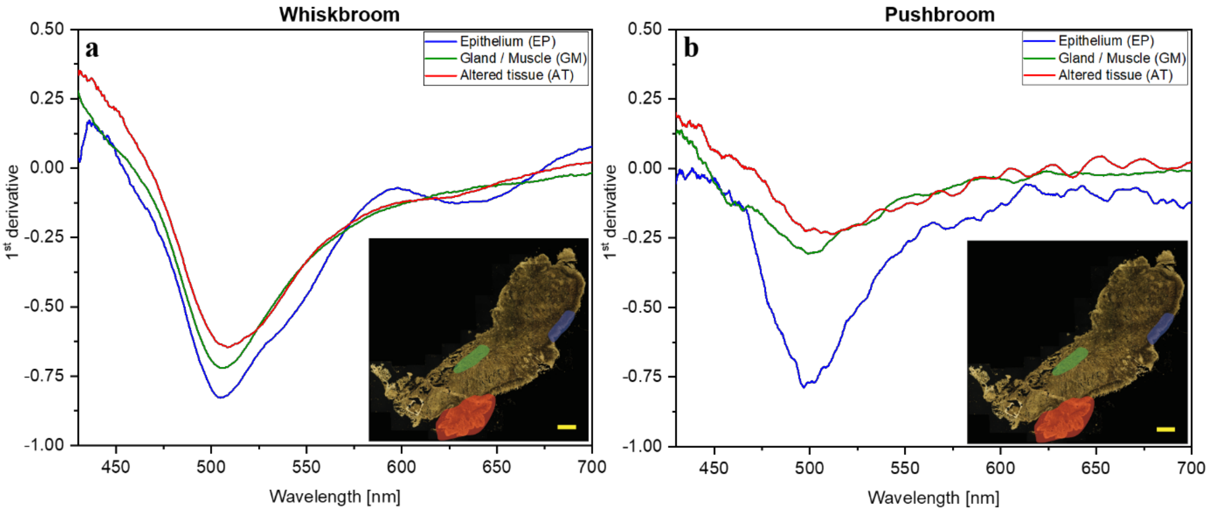

Fig. S1 *Exemplary preprocessed ELS spectra of EP (blue), GM (green) and AT (red) tissue used for Whiskbroom (a) and Pushbroom (b) PCA-DA model formation. By applying the described preprocessing steps (moving average smoothing with 47 segment points, baseline offset correction, gap derivation (1st derivative, gap size: 15 pts.) and range-normalization), the periodic scattering structure can be extracted and become more clearly visible. Although spectra were acquired in a wavelength range of 412-975 nm for Whiskbroom and 398-715 nm for Pushbroom detection, only the wavelength region from 430-700 nm is shown for reasons of comparability. Each preprocessed spectrum corresponds to one tissue type, exemplarily indicated by the colored ellipses in the DF image of one tissue section. Yellow scale bar: 1000 µm*

For the sake of completeness, the respective 2D scores plots of PC3 vs. PC2 and PC4 vs. PC3 for the Whiskbroom and Pushbroom PCA are displayed in Fig. S2 and S3. Corresponding loadings plots for both PCAs are additionally illustrated.

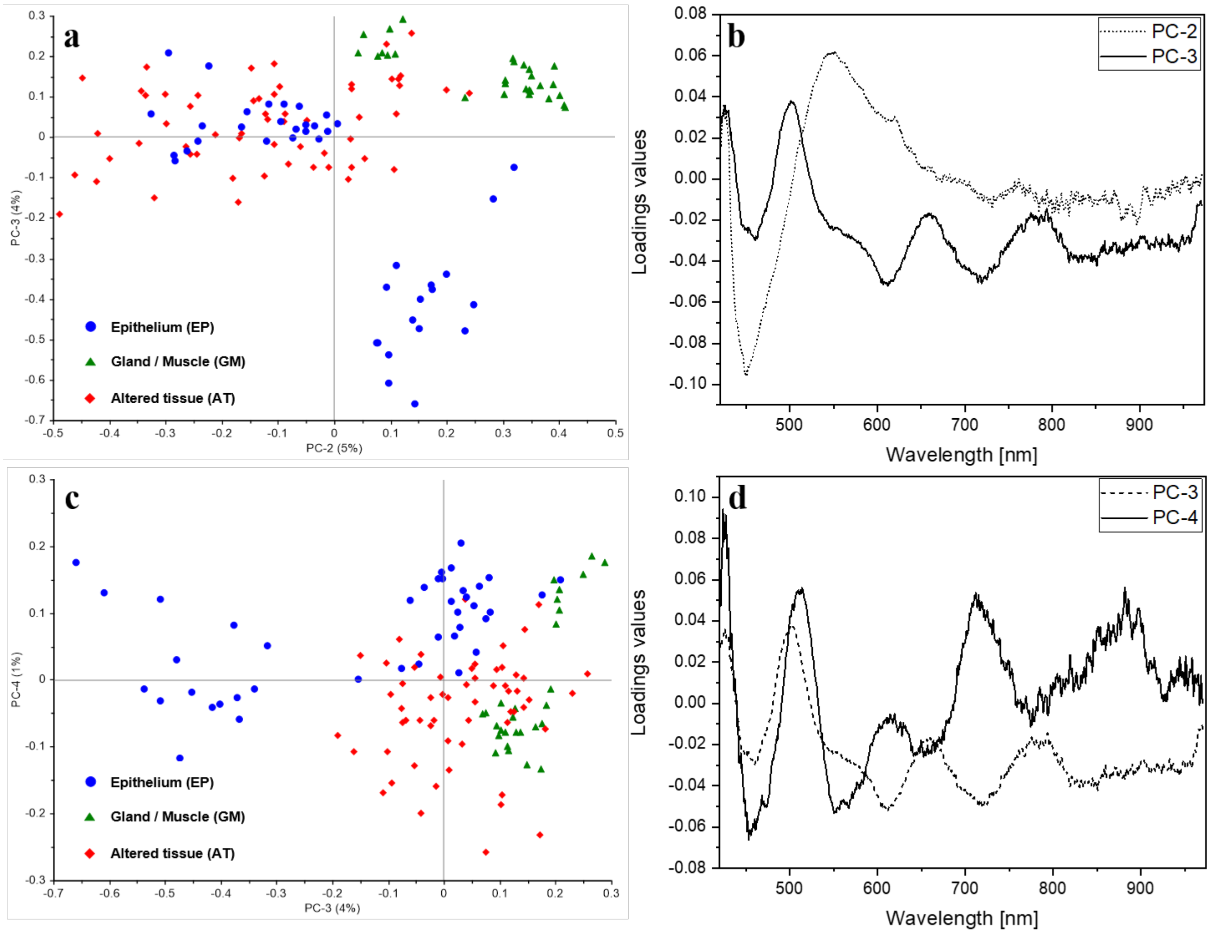

Fig. S2 *2D scores plot of the Whiskbroom PCA for the differentiation of EP (blue), GM (green) and AT (red). In a: The 2D scores plot of PC3 against PC2 reveals the distinct separation of the GM and AT groups and a splitting of the EP cluster in two, of which one is completely overlapping with the AT cluster. A further splitting of the GM into two subpopulations is also observable. PC2 explains 5 % whereas PC3 represents 4% of the overall variance. In b: Corresponding loading plots for PC2 and PC3 of the calculated PCA. In c: The 2D scores plot of PC4 against PC3 still reveals a splitting of the EP and GM cluster into two subgroups, respectively. However, no overlapping of the EP and AT conglomerations is visible, but instead one of the GM subgroups extend into the AT cluster. In this case, PC3 still represents 3 % and PC4 describes 1 % of the total variance. In d: Corresponding loading plots for PC3 and PC4 of the calculated PCA*

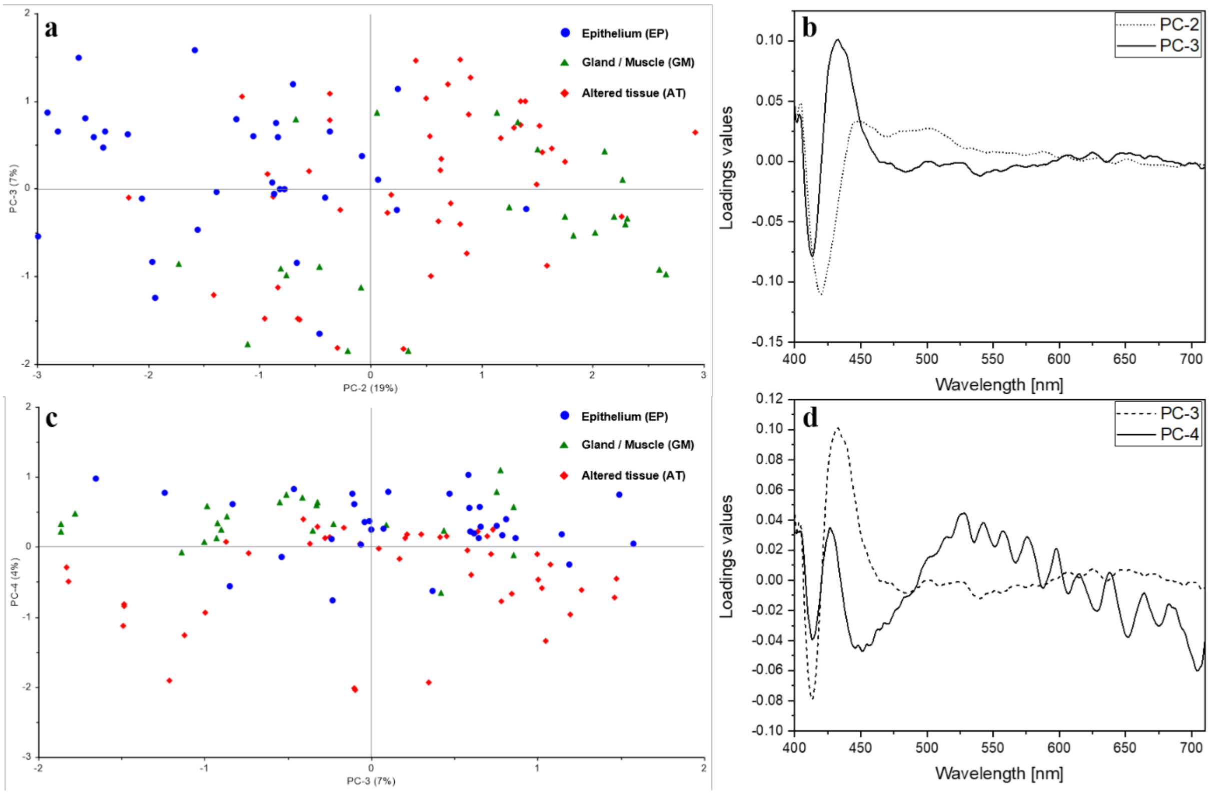

Fig. S3 *2D scores plot of the Pushbroom PCA for the differentiation of EP (blue), GM (green) and AT (red). In a: The 2D scores plot of PC3 against PC2 reveals no separation of the GM, AT and EP groups. All three tissue clusters are significantly overlapping and show no separation tendency at all. As demonstrated above, a splitting of the GM group is recognized. PC2 explains 19 % whereas PC3 represents 7% of the overall variance. In b: Corresponding loading plots for PC2 and PC3 of the calculated PCA. In c: The 2D scores plot of PC4 against PC3 only reveals a slight trend of tissue type segregation. The AT cluster mainly relates to negative score vales for PC4 whereas the EP and GM groups primarily obtain positive score values for PC4. PC3 still represents 7 % and PC4 describes 4 % of the total variance. In d: Corresponding loading plots for PC3 and PC4 of the calculated PCA*

The calculation of the overall accuracy, sensitivity, specificity and precision is based on the classification outcome of the Whiskbroom and Pushbroom PCA-DA models in terms of true positive and negative cases as well as false positive and negative ones. All results are summarized in the confusion matrix for each model, which are depicted in Tab. S1 and S2.

Tab. S1 *Confusion matrix of the Whiskbroom PCA-DA model. Each column of the displayed matrix represents the number of ELS spectra included in the Whiskbroom model, which are 58 AT, 28 GM and 41 EP spectra. Each matrix row, however, lists the model's prediction results of the respective tissue type group. For the Whiskbroom model, 41 EP out of 41 EP spectra and 28 GM out of 28 GM spectra were correctly assigned. Compared to that, 56 AT were matching the total amount of 58 AT spectra whereas 2 AT were attributed to EP.*

**Confusion matrix Whiskbroom PCA-DA**		**Actual**		
		Altered tissue (AT)	Gland /Muscle (GM)	Epithelium (EP)
**Predicted**	Altered tissue (AT)	56	0	0
	Gland / Muscle (GM)	0	28	0
	Epithelium (EP)	2	0	41

Tab. S2 Confusion matrix of the Pushbroom PCA-DA model. Each column of the displayed matrix represents the number of ELS spectra included in the Pushbroom model, which are 44 AT, 24 GM and 32 EP spectra. Each matrix row, however, lists the model's prediction results of the respective tissue type group. For the Pushbroom model, 44 AT out of 44 AT spectra were correctly assigned. Compared to that, 23 GM out of 24 GM and 32 EP out of 33 EP were correctly matching whereas 1 GM and 1 EP were attributed to either EP or GM.

**Confusion matrix Pushbroom PCA-DA**		**Actual**		
		Altered tissue (AT)	Gland /Muscle (GM)	Epithelium (EP)
**Predicted**	Altered tissue (AT)	44	0	0
	Gland / Muscle (GM)	0	23	1
	Epithelium (EP)	0	1	32

The total variances of the PCA-DA models are expressed as an accumulation of the individual PC-variances. For each PC, the calculated variance identifies how well the investigated samples are described by the PC and thus how much variation in the data can be explained by the corresponding model. Since the model formation is based on the calibration data set, a calibration variance of the model can be defined. A model testing, however, is accomplished by a model validation and thus a validation variance can additionally be calculated. In case both parameters do not exhibit a similar trend, the calibration or testing data might not be representative. In Fig. S4, a summary of the total variances is illustrated for each PCA-DA model.

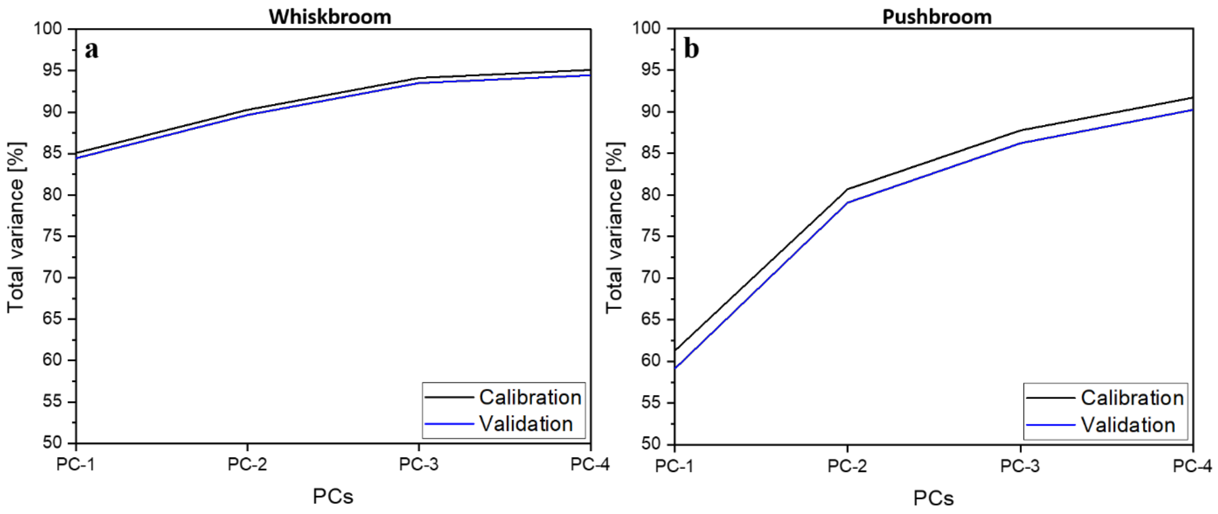

Fig. S4 Overview of the total variances for PC-1 to PC-4 of the Whiskbroom (a) and Pushbroom (b) PCA-DA model, respectively. In a: PC-1 of the Whiskbroom model already explains 85 % of the data's total variance which finally adds up to 95 % considering PC-1 to PC-4 (a). In b: For the Pushbroom model, PC-1 illustrates 61 % of the total variance whereas the accumulation of all four PCs describes 90 % (b). The calibration and validation variance reveal almost identical curve shapes for both models and thus calibration as well as testing data sets are well explained by the two models.
